# Early Manifestation of Clozapine-Induced Cardiotoxicity: Detection, Pathophysiology, and Management

**DOI:** 10.7759/cureus.27202

**Published:** 2022-07-24

**Authors:** Steven Hamilton, Rana A Tauseen, Natale Wasef, Andreas Wolf

**Affiliations:** 1 Internal Medicine, Jersey Shore University Medical Center/Saint Francis Medical Center Program, Trenton, USA; 2 Cardiology, Mercer Bucks Cardiology/Saint Francis Medical Center, Trenton, USA

**Keywords:** c-reactive protein (crp), clozapine, cardiotoxicity, schizoaffective, cardioselective beta blockers, left ventricular systolic dysfunction, sinus tachycardia

## Abstract

Schizoaffective disorder, bipolar type is a chronic mental health disorder that may manifest as mania. Clozapine is effective in treating acute mania and in achieving mood stabilization. However, on rare occasions, the use of clozapine has been associated with cardiotoxicity. Here, we present a case of a 31-year-old man who at baseline is known to have schizoaffective disorder, bipolar type, and cannabis dependence and was admitted to our hospital with a psychotic relapse. He was treated with clozapine, uptitrated to a maximum daily dose of 200mg twice daily by day 10. Thereafter he became febrile and experienced malaise, myalgias, and chest pain. He was noted on electrocardiogram to have sinus tachycardia without ischemic changes. In this context, he had a troponin leak, increased white blood cell count, serologies and cultures were negative and chest x-ray revealed no acute disease of the chest. Due to the suspicion of clozapine-induced cardiotoxicity, a transthoracic echocardiogram was done, which revealed mildly depressed left ventricular (LV) systolic function without pericardial effusion. Thereafter, clozapine was withdrawn and switched to lithium. Additionally, the cardioselective, metoprolol tartrate was initiated. Within 36-48 hours, he had resolution of symptoms and remained cardiovascularly stable. Clozapine uncommonly causes cardiotoxicity and early features may be non-specific. Awareness of this and recognizing early features aids in reducing the associated cardiovascular morbidity and mortality.

## Introduction

Clozapine is an atypical antipsychotic drug used for the treatment of schizoaffective disorder, bipolar type. It is famously associated with the frequently tested board examination adverse effect of agranulocytosis. However, in the same vein, there is an FDA black box warning about the cardiovascular adverse effect profile, which includes cardiomyopathy and fatal myocarditis. Clozapine-induced early cardiotoxicity has non-specific clinical features such as chest pain, dyspnea, fever, and flu-like symptoms which represent a diagnostic challenge, requiring vigilance and a high index of clinical suspicion. With respect to the diagnostic workup, the transthoracic echocardiogram is a useful tool to aid in the diagnosis of clozapine-induced early cardiotoxicity which may reveal a depressed left ventricular systolic function. Additionally, typically there will be associated increases in cardiac biomarkers and inflammatory markers. Here, we report a case of a 31-year-old man who started on clozapine with subsequent development of cardiotoxicity. Our focus is the recognition of pathophysiology and management of clozapine-induced early cardiotoxicity.

This article was previously presented as a poster abstract in the 2021 ACPNJ Virtual Abstract Competition on March 11-13, 2021.

## Case presentation

**Figure 1:**
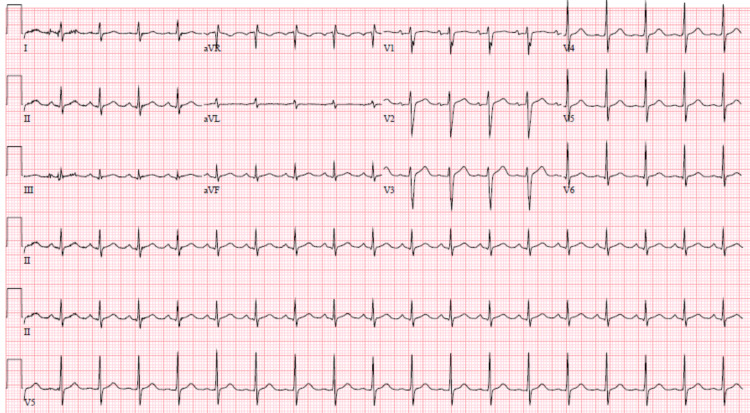
Electrocardiogram showing sinus tachycardia, without ischemic changes.

A 31-year-old man who at baseline is known to have schizoaffective disorder, bipolar type and cannabis dependence was brought to our hospital by law enforcement because of delusional thoughts. He was brought in after being charged with criminal mischief due to aggressive behavior. He endorsed non-compliance with his quetiapine 800mg at bedtime because he believed he was cured of schizoaffective disorder. He admitted to regular cannabis use and did not endorse suicidal ideations. He was admitted to our psychiatry floor with a diagnosis of psychotic relapse of schizoaffective disorder, bipolar type, and cannabis use disorder. He was initiated on clozapine 12.5mg orally twice daily which was then uptitrated to 25mg twice daily on day three of admission. Clozapine was then uptitrated by 12.5mg per dose every two days up to a maximum of 200mg twice daily on day 10, which was maintained thereafter. On day 14, he was noted to have a temperature of 37.8°C which achieved a maximum temperature of 39.3°C on day 16, associated with malaise, generalized myalgias, and chest pain. He did not endorse shortness of breath, leg swelling, coughing, sore throat, nausea or vomiting, headache, abdominal pain, or dysuria. Other vital signs included blood pressure of 106/50 mmHg and heart rate of 113 beats per minute. Physical examination revealed a regular and rapid pulse, S1 and S2, without rubs, murmurs, or gallops. There was no jugular venous distension. His lungs were clear to auscultation. Laboratory studies revealed highly sensitive troponin I 0.19 ng/mL, c-reactive protein (CRP) 10.5, white blood cell (WBC) count 10.5, and platelet count 126 (Table 1). A 12-lead electrocardiogram (EKG) revealed sinus tachycardia, rate at 111 beats per minute, without ischemic changes at which time he was transferred to the medical floor (Figure 1).

**Table 1:** Biochemical parameters of the patient. CRP: c-reactive protein

Day of admission	Troponin (ng/mL)	Reference values (troponin) (ng/mL)	WBC (10^3^/uL)	Reference values (WBC) (10^3^/uL)	CRP (mg/dL)	Reference values (CRP) (mg/dL)
Day 1	-	-	7.1	3.8-10.2	-	-
Day 16	0.19	<0.03	10.5	3.8-10.2	10.54	0.02-1.00
Day 18	0.12	<0.03	8.3	3.8-10.2	9.0	0.02-1.00
Day 19	0.07	<0.03	6.5	3.8-10.2	5.4	0.02-1.00

Blood culture showed no growth. Influenza A and B antigens, Streptococcus group A antigen, and SARS-CoV-2 polymerase chain reaction (PCR) were negative. HIV antibody and antigen were nonreactive. Urine drug screen was positive for marijuana. CXR revealed no acute disease findings. Given the non-specific clinical picture and his troponin leak cardiology was consulted and a presumptive diagnosis of clozapine-induced cardiotoxicity, with consideration of related cardiomyopathy, and possible myocarditis was made. Due to the absence of EKG abnormalities and the troponin elevation being less than would be expected in the setting of myocarditis, a transthoracic echocardiogram was ordered. The echocardiogram revealed decreased left ventricular systolic function and an ejection fraction of 40-50% without regional wall motion abnormalities.

As a result of this high suspicion for clozapine-induced cardiotoxicity (rule out cardiomyopathy or myocarditis), the last dose of clozapine 100mg was administered on the night of day 15 of admission and he was switched to lithium carbonate 600mg orally twice daily. Additionally, he was started on metoprolol tartrate 12.5mg orally twice daily on day 16 and monitored via continuous telemetry. The patient subsequently became afebrile 36-48 hours after discontinuation of the clozapine. There was resolution of the chest pain, malaise, and myalgias, without development of shortness of breath or lower leg swelling. He remained cardiovascularly stable and was transferred back to the psychiatry floor for the remainder of his hospitalization, where he did well and was subsequently discharged in a stable condition.

## Discussion

Clozapine is the medication of choice in treatment‐resistant psychotic disorders including schizoaffective disorder. Agranulocytosis is the adverse effect most commonly taught and mentioned in the medical literature. However, cardiotoxicity may uncommonly occur and at times can be life-threatening. The development of cardiotoxicity as a result of clozapine therapy generally occurs within the first two to eight weeks of therapy [1]. At the time of diagnosis, the mean age of patients has been reported as 33.5 years with a mean dose of clozapine 360mg/day [2]. Other researchers have found that the dose at the onset of symptoms and signs was between 50mg/day and 600mg/day while the median was 250mg/day in the 53 cases where patient-specific data were available. These findings are similar to the findings of several summated reports and reviews [3]. Our patient was on a maximum of 200mg/day at the time of symptom onset. According to Patel et al., there are no established specific risks [4]. As such besides exposure to clozapine, there are no predisposing factors that currently suggest who may experience clozapine-induced cardiotoxicity. Importantly, there is no classic presentation of clozapine-induced cardiotoxicity. Symptoms exist on a spectrum, including chest pain, fever, shortness of breath, palpitations, and leg swelling. In one systematic review by Bellissima et al., they found that fever (67%) and tachycardia (58%) were two of the most common clinical features [3]. Our patient manifested fever and tachycardia early on, with an increased cardiac biomarker level, highly sensitive troponin I of 0.19ng/mL. This troponin leak reflects the myocardial inflammatory changes that take place in the setting of clozapine-induced cardiotoxicity. Notably, as he clinically improved this troponin level decreased accordingly.

Clozapine-induced increase in serum catecholamines increases the myocardial oxygen demand both directly and indirectly via direct myocardial stimulation and increasing cardiac afterload and decreasing myocardial oxygen perfusion. This rise in serum catecholamines correlates with the degree of myocardial inflammation. Increased catecholamines stimulate the renin-angiotensin-aldosterone system leading to further increase in the cardiac afterload [5]. Interestingly, studies have found that chronic cannabis use is associated with increased catecholamine neurotransmitters [6,7]. Our patient being a chronic and frequent cannabis user could have potentiated the aforementioned mechanism and contributed to the early manifestation of cardiotoxicity. Concurrently, increased cardiac afterload with decreased perfusion, myocardial ischemia, and increased production of free reactive oxygen species leads to an increase in myocardial lipid peroxidation, inflammation, and cell injury. 

Another mechanism that has been proposed is that of a type I IgE-mediated acute hypersensitivity reaction. In a review conducted of 47 cases of clozapine-induced myocarditis, the development of eosinophilia suggesting IgE-mediated hypersensitivity reaction has been documented in approximately 66% of cases [8]. Though our patient did not have an absolute increase in eosinophils above the normal range, his values did increase by a factor of six at the time of symptom onset. When endomyocardial biopsies have been done, eosinophilic myocardial infiltrates have been found [9]. Activated eosinophils promote tissue injury and necrosis through the production and release of reactive oxygen metabolites and cytotoxic proteins (including myeloperoxidase) into the extracellular fluid [5]. Of note, our patient developed leukocytosis with neutrophil predominance and neutrophil migration has been used as an index of increased cardiac myeloperoxidase levels in clozapine-induced cardiotoxicity. A third pathophysiological consideration is that clozapine is known to undergo bioactivation in myocardial tissue to a reactive nitronium ion metabolite. This nitronium ion stimulates cellular injury, lipid peroxidation, and free radical production [5]. Additionally, it binds with myocardial proteins, forming antigenic complexes which stimulate the immune response and macrophages. This complex leads to myocardial cell damage via the release of free radicals and the activation of proinflammatory cytokines such as tumor necrosis factor-alpha (TNF-alpha). TNF-alpha subsequently attracts leukocytes, enhancing the generation of reactive species leading to further myocardial insult [9].

Biochemical testing to evaluate for potential myocardial damage with elevated cardiac biomarkers is essential. This was reflected in our patient as he initially had a troponin leak at the time of symptom onset. Trending levels of inflammatory markers such as erythrocyte sedimentation rate (ESR) and CRP have been suggested as ways to document recovery [8]. In the case of our patient, we trended CRP for 72 hours from symptom onset. The CRP was elevated at the time of symptom onset and then the level decreased over the subsequent 48 hours. Additionally, electrocardiogram (EKG) and chest x-ray (CXR) are required for workup. In our patient, CXR did not reveal acute disease of the chest including cardiomegaly and EKG revealed sinus tachycardia which was the most common manifestation (37%) in the systematic review by Bellissima et al. [3]. It is important to assess left ventricular function in the setting of possible clozapine-induced cardiotoxicity and echocardiography is diagnostically useful here. Our patient underwent transthoracic echocardiography which revealed mildly decreased left ventricular systolic function and an approximated ejection fraction between forty to fifty percent, without regional wall motion abnormalities. If this was acute myocarditis we would typically expect the transthoracic echocardiogram to include regional or global left ventricle or biventricular systolic dysfunction with normal wall thickness. Ideally, evaluation of coronary anatomy through cardiac catheterization would have been helpful to rule out ischemic disease. However, this was deemed unnecessary due to the temporal relationship of the clozapine to his clinical features and biochemical results and the fact that he was actively psychotic. We are compelled to highlight that it is unlikely that our patient had established florid clozapine-induced cardiomyopathy or myocarditis, as there was no shortness of breath, fatigue, jugular venous distension, or peripheral edema. Chest x-ray was unremarkable as well and transthoracic echocardiogram was without severe left ventricular (LV) dysfunction as previously mentioned. In this context, we confidently concluded that our patient had early clozapine-induced cardiotoxicity which was detected before significant decline in cardiac function.

Definitive diagnosis is made by utilization of endo-myocardial biopsy, which was not available in our community hospital. In the absence of endo-myocardial biopsy, a cardiac MRI is highly useful, which was also not available in our community hospital. As such, we recognize and acknowledge the limitations of not having cardiac catheterization, cardiac MRI, or endomyocardial biopsy for a definitive diagnosis. That being said, most cases of clozapine-induced cardiotoxicity are not definitely diagnosed. In a patient with clozapine-induced early cardiotoxicity, there should be symptomatic and biochemical resolution with withdrawal of the drug, which was evident in our patient. A cardioselective beta blocker, metoprolol tartrate was utilized to support myocardial function and treat his tachycardia during the myocardial insult. Discontinuation of clozapine leads to cardiac functional recovery with a direct correlation between degree of compromised systolic function and degree of recovery [5]. In our patient’s case, the degree of compromised systolic function was mild and as such we expected a complete recovery. It would likely be beneficial to repeat this patient’s transthoracic echocardiogram, in three to six months to demonstrate recovery of his left ventricular systolic function. Along the same line, it is important to recall that recognizing clozapine-induced cardiotoxicity and its subsequent management is not without risks to the patient. For this reason, the medication management benefits from psychiatry involvement to prevent relapse of an acute psychiatric episode, as was done in this case, to reduce patient harm. Under the guidance of psychiatry, he was transitioned to lithium carbonate with improvement.

## Conclusions

The correlation of clinical, biochemical, and echocardiographic features followed by resolution and improvement with withdrawal of clozapine supports a diagnosis of clozapine-induced early cardiotoxicity. The use of biomarkers, transthoracic echocardiogram, and the temporal relationship to clinical features aid in making this diagnosis in the absence of resources such as endomyocardial biopsy and cardiac MRI. Additionally, we agree with prior authors who propose that it may be beneficial to monitor patients while on clozapine. Monitoring may include doing weekly biomarker monitoring and perhaps even a baseline transthoracic echocardiogram before initiating clozapine therapy given the significant cardiovascular outcomes that may occur.

## References

[1] Curto M, Girardi N, Lionetto L, Ciavarella GM, Ferracuti S, Baldessarini RJ (2016). Systematic review of clozapine cardiotoxicity. Curr Psychiatry Rep.

[2] Alawami M, Wasywich C, Cicovic A, Kenedi C (2014). A systematic review of clozapine induced cardiomyopathy. Int J Cardiol.

[3] Bellissima BL, Tingle MD, Cicović A, Alawami M, Kenedi C (2018). A systematic review of clozapine-induced myocarditis. Int J Cardiol.

[4] Patel RK, Moore AM, Piper S (2019). Clozapine and cardiotoxicity - a guide for psychiatrists written by cardiologists. Psychiatry Res.

[5] Abdel-Wahab BA, Metwally ME (2014). Clozapine-induced cardiotoxicity in rats: Involvement of tumour necrosis factor alpha, NF-κβ and caspase-3. Toxicol Rep.

[6] Fitzgerald PJ (2013). Elevated norepinephrine may be a unifying etiological factor in the abuse of a broad range of substances: alcohol, nicotine, marijuana, heroin, cocaine, and caffeine. Subst Abuse.

[7] Cohen K, Weizman A, Weinstein A (2019). Modulatory effects of cannabinoids on brain neurotransmission. Eur J Neurosci.

[8] Ronaldson KJ, Taylor AJ, Fitzgerald PB, Topliss DJ, Elsik M, McNeil JJ (2010). Diagnostic characteristics of clozapine-induced myocarditis identified by an analysis of 38 cases and 47 controls. J Clin Psychiatry.

[9] Shin WS, Szuba A, Rockson SG (2002). The role of chemokines in human cardiovascular pathology: enhanced biological insights. Atherosclerosis.

